# Social Investigation and Long-Term Recognition Memory Performance in 129S1/SvImJ and C57BL/6JOlaHsd Mice and Their Hybrids

**DOI:** 10.1371/journal.pone.0054427

**Published:** 2013-01-16

**Authors:** Jana Hädicke, Mario Engelmann

**Affiliations:** 1 Otto-von-Guericke-Universität, Institut für Biochemie und Zellbiologie, Magdeburg, Germany; 2 Center for Behavioral Brain Sciences, Magdeburg, Germany; University of Illinois-Chicago, United States of America

## Abstract

When tested for their behavioural performance, the mixed genetic background of transgenic mice is a critical, but often ignored, issue. Such issues can arise because of the significant differences in defined behavioural parameters between embryonic stem cell donor and recipient strains. In this context, the commonly used stem cell donor strain ‘129’ shows ‘deficits’ in different paradigms for learning and long-term memory. We investigated the long-term social recognition memory performance and the investigative behaviour in commercially available 129S1/SvImJ and C57BL/6JOlaHsd mice and two F1-hybrids (129S1/SvImJ×C57BL/6JOlaHsd) by using the social discrimination procedure and its modification, the volatile fraction cage (VFC). Our data revealed an unimpaired olfactory long-term recognition memory not only in female and male 129S1/SvImJ and C57BL/6JOlaHsd mice but also in the two hybrid lines (129S1/SvImJxC57BL/6JOlaHsd) when the full ‘olfactory signature’ of the ‘to-be-recognized’ conspecific was presented. Under these conditions we also failed to detect differences in the long-term recognition memory between male and female mice of the tested strains and revealed that the oestrus cycle did not affect the performance in this memory task. The performance in the VFC, based only on the volatile components of the ‘olfactory signature’ of the ‘to-be-recognized’ conspecific, was similar to that observed under direct exposure except that females of one F1 hybrid group failed to show an intact long-term memory. Thus, the social discrimination procedure allowing direct access between the experimental subject and the stimulus animal(s) is highly suitable to investigate the impact of genetic manipulations on long-term memory in male and female mice of the strain 129S1/SvImJ, C57BL/6JOlaHsd and 129S1/SvImJxC57BL/6JOlaHsd hybrids.

## Introduction

There is increasing evidence that inbred mouse strains differ in their behavioural profile. Indeed, various studies have reported significant differences in defined behavioural parameters between different commercially available mouse strains including the C57BL/6J strain and inbred ‘129’ substrains [1]–[5]. In particular substrains of the latter, the commonly used embryonic stem cell donor strain, show impaired learning and long-term memory performance [5]–[7], that is paralleled by alterations in brain circuitry [8]–[9]. Thus, although the design of molecular tools for manipulating the mouse genome is continually progressing, the mixture of genetic material from different mouse strains remains critical [10]–[13]. To cope with these mouse strain-differences, guidelines were proposed to enable the use of mutant mice in tasks in which 129 mice show a very poor learning and memory performance (e.g. numerous backcrosses to reduce the genetic material of the 129 strain) and – very recently – a report suggested using animals of the C57BL/6N strain as stem cell donors [14].

The alternative approach would employ a learning and memory task in which animals of the 129 strain show an unimpaired behavioural performance similar to other commonly used mouse strains. Tests that investigate the olfactory cued “social recognition memory“ may provide such an alternative. Typically, the olfactory cues are provided by the odour emitted from a given animal, often called ‘olfactory signature’, which includes both volatile (which are detectable over a distance) and non-volatile components (detectable by direct body contact only) [15]–[17]. Such tests rely on the animals’ intrinsic motivation to acquire the ‘olfactory signature’ of a conspecific and that unfamiliar items will be investigated over familiar items [18]. Therefore, a significantly longer investigation duration towards a novel versus a previously encountered conspecific is taken as an evidence for successful recognition. However, a recent study reports impaired social memories in 129P2 inbred mice [19] when tested in the “social recognition procedure” originally established in 1987 for testing social memory in rats [20].

The social discrimination test [21]–[22] was introduced to overcome some technical limitations of the social recognition procedure and offers another possibility to analyse olfactory-cued social recognition memory in mice. The main modification concerns the simultaneous presentation of the previously encountered with a new stimulus animal which enables a direct judgement of the recognition/discrimination abilities of the experimental subject in a single session. In the original version of this test the experimental subject gets direct access to the stimulus animals, thereby enabling the acquisition of the full ‘olfactory signature’ (including both the volatile and non-volatile components). In a recently published modification of this procedure the access can be restricted to the volatile components only by using the volatile fraction cage (VFC) [23]–[24].

The present study was designed to examine the investigative behaviour and long-term social recognition memory performance in 129S1/SvImJ mice compared to C57BL/6JOlaHsd animals using the social discrimination task and the VFC. We also established and tested two F1-hybrid lines by mating females or males of the 129S1/SvImJ strain with male and female, respectively, C57BL/6JOlaHsd mice. The use of mothers from the different strains was designed to characterize possible influences of maternal behaviour on the behavioural performance of the heterozygous offspring when tested as adults for their social memory. F1 hybrids provide a model of a mixed genetic background which may easily reveal a potential memory distracting impact in this model. Other authors reported that F1 hybrids of 129 and C57B6 mice show dramatic changes in the ataxia index [25]. The present study extended these findings by including analysis of the olfactory recognition memory performance in female mice of all lines listed.

## Materials and Methods

### Animals

Adult male and female 129S1/SvImJ (male: n = 23, female: n = 20; The Jackson Laboratory, Charles River, Sulzfeld, Germany) and C57BL/6JOlaHsd mice (male: n = 24, female: n = 26; Harlan-Winkelmann, Borchern, Germany;) and two F1-hybrid lines were used as experimental subjects. The F1-hybrids were created by mating female 129S1/SvImJ with male C57BL/6JOlaHsd (**Hyb1;** male: n = 20, female: n = 18) and female C57BL/6JOlaHsd with male 129S1/SvImJ (**Hyb2;** male: n = 20, female: n = 20). All animals were housed in groups of three to six per cage (size 20×37×15 cm) under standard laboratory conditions (temperature: 23±1°C; humidity: 60±5%; 12 h light/12 h dark cycle with lights on at 07∶00) with free access to food and water. All tested mice were at an age of 8–16 weeks during testing and sexually naive. Juvenile mice of both sexes (25–35 days old; C57BL/6JOlaHsd strain) were used as olfactory stimuli. Previous extensive studies in our laboratory have shown that neither the sex nor the defined age of the juveniles significantly affects social recognition memory performance in C57BL/6JOlaHsd mice [26]. If not stated otherwise, stimulus animals were kept in groups of 3–5.

#### Ethics statement

All experimental manipulations were approved according to German and European legislation by the *Landesverwaltungsamt Sachsen-Anhalt, Referat Verbraucherschutz, Veterinärangelegenheiten, Halle,* Germany (approval number: 203.k-42502-2-992UniMDG).

### Determination of the Stage of the Oestrus Cycle

As vaginal stimulation prior to a behavioural experiment has been shown to influence learning and memory performance in female rodents [27]–[30], vaginal smears were obtained after the end of choice in each experiment. Sterile Q-tips were immersed in physiological saline and gently inserted into the vaginal tract to remove loose cells. Smears were transferred onto a glass slide, air dried and Nissl stained. Based on the vaginal smear cytology mice were assigned to two groups: oestrus and non-oestrus during choice. According to the previously described impact of the oestrus cycle primarily on memory acquisition we extrapolated the cycle phase during sampling from the samples collected after choice by estimating a duration of the oestrus cycle of 24 h [31]. Thus, females showing an oestrus smear during choice were estimated as being tested in prooestrus during sampling (24 h exposure interval). In contrast, smears not showing typical oestrus signs suggested that the females were tested in non-prooestrus (including dioestrus, metoestrus, oestrus) during sampling.

### Social Discrimination Test and the Volatile Fraction Cage (VFC)

A detailed description of both procedures is provided by Engelmann et al. (2011) including the detailed description of the sequence in which the stimulus animals are used during testing. Briefly, the tests consist of two sessions (sampling and choice) during which given juveniles are exposed as stimulus animals to the experimental subjects for 4 min during each session. Previous testing revealed that both male and female juveniles are suitable to serve as stimulus animals [26]. Two hours before starting the test experimental subjects were separated by transferring them into small cages (home cage; 14×20×15 cm). When using the VFC there was an additional separation time of the experimental subject in the VFC (10 min) before starting the experiment. During the first encounter (sampling; performed during the light phase between 8.00 h and 13.00 h), the ‘to-be-recognized’ juvenile was exposed to the experimental subject for acquiring the stimulus animals’ ‘olfactory signature’. Then, the juvenile was removed, kept individually in a fresh cage with food and water *ad libitum* for the exposure interval of 24 h. During the second encounter (choice), the familiar juvenile was re-exposed to the experimental subject together with an additional, previously not encountered (novel) juvenile. The monitored olfactory investigatory behaviour of the experimental subject toward the stimulus animals during choice serves as an index for recognition: a significantly longer investigation duration of the novel versus the previously encountered conspecific is taken as an evidence for successful recognition. To allow for easy identification of the juveniles by the observer during the choice, tail marking was used (Edding 30 permanent marker, red or green, Edding AG, Germany), which does not affect social discrimination [26]. After conclusion of each test the animals were put back in the original groups until the next test.

Two series of experiments were performed. All experimental subjects (the number of animals per group is given in the respective figures) were first tested in the first experimental series and one week later in the second experimental series. Another week later the animals were tested in the open field (for the results see **Fig. S1** and **Table S1**). During the **first series,** the social recognition performance was monitored using the social discrimination procedure with direct access to the stimulus animals during both sampling and choice. During the **second** **series,** the social discrimination procedure was modified by employing the VFC (indirect contact) [26]. Because of computer failures during the measurement of choice one male mouse was excluded in the first experimental series, and five male mice in the second series.

#### First series: Direct stimulus animal presentation during both sampling and choice

**Figure 1:**
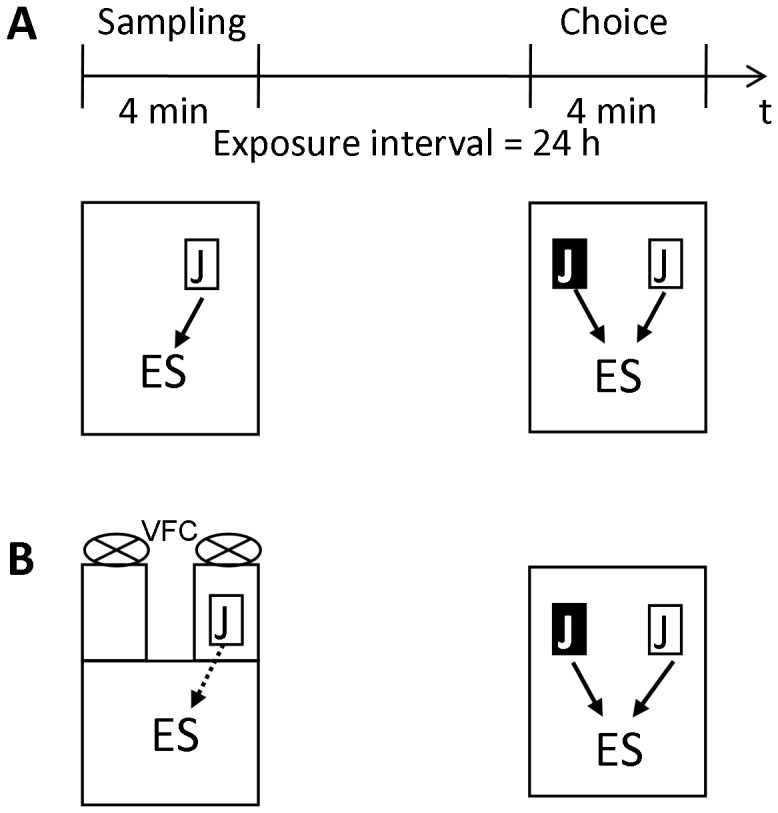
Design of the two series of experiments in which the social discrimination performance was tested. A given juvenile (J in white box) was exposed to the experimental subject (ES) during a 4-min sampling session. This juvenile was removed and, after the exposure interval of 24 h, re-exposed to the experimental subject during the choice session, together with a different, not previously encountered juvenile (J in black box). **A:** Direct exposure of the juvenile(s) in the home cage of the experimental subject with direct interaction between experimental subject and stimulus animal(s) during both sampling and choice allowing the acquisition of the full olfactory signature of the juvenile(s) (black arrow(s)). **B:** Sampling was performed in the volatile fraction cage (VFC, presentation of only the juvenile’s volatile components of the ‘olfactory signature’ = hatched arrows). Choice was performed in the home cage of the experimental subject allowing direct interaction between the animals and, thus, the acquisition of the full olfactory signature (black arrows).

Sampling and choice took place in the home cage of the experimental subject under unrestricted conditions **(** **Fig. 1A**). Thus, the experimental subject had direct contact to the juvenile(s) and access to the full juveniles’ ‘olfactory signature’ in both sessions. During each session, the duration of investigatory behaviour of the experimental subject towards each juvenile (licking, sniffing) was monitored separately by a trained observer unaware of the animals genotype and sex by pressing pre-set keys on a keyboard and a computer program.

#### Second series: Sampling performed in the VFC, direct stimulus animal presentation during choice

During the second series, choice was performed in the experimental subject’s home cage as indicated above. Sampling was performed in the VFC that separated the experimental subject from the stimulus juveniles. As described in detail elsewhere [26], a fan behind the juvenile produced a constant air flow that carried only the volatile components of the juvenile’s ‘olfactory signature’ to the experimental subject (**Fig. 1B**). Thus, the experimental subject gained access to only the volatile components of the juvenile’s ‘olfactory signature’ during sampling, but was allowed to acquire the full ‘olfactory signatures’ of the familiar and the novel juvenile during choice. In the VFC, active sniffing of the experimental subject toward the air streams containing the volatile information of the respective juvenile was measured as indicated above. Acquisition of the volatile component of the olfactory signature in the VFC is subsequently termed ‘indirect sampling’ whereas direct exposure of the stimulus animal to the experimental subject ‘direct sampling’.

### Statistical Analyses

Statistical analysis was performed using GB-STAT 6.0 (Dynamic Microsystems, Silver Springs, MD, U.S.A.) or GraphPad Prism 4.01 (GraphPad Software, San Diego, CA, U.S.A.). Data obtained from the social discrimination test are presented as means +/− SEM and were used to calculate the recognition index (RI), which is a measure of the recognition ability [32]. The RI was calculated on the parameters measured during choice and is the quotient of the investigation duration of the novel juvenile divided by the sum of the investigation durations of familiar and novel juvenile×100. Chance performance is 50. A RI significantly above 50 indicates successful recognition of the familiar juvenile [26]. The RIs were analyzed using a one sample t-test against a theoretical mean of 50 and two-way ANOVA (genotype×sex). Additionally, investigation durations towards the novel and the previously encountered juveniles during choice were compared using the paired t-test [26]. For analysing the influence of genotype and sex on the sampling investigation two-way ANOVAs (genotype×sex) were carried out for the two experimental series. For analyzing the influence of juvenile’s sex, a three-way ANOVA was used (genotype×experimental subject’s sex×juvenile’s sex). To analyse the impact of the oestrus cycle on the performance of the female experimental subjects RIs were submitted to a two-way ANOVA (experimental series×oestrus stage (i.e. prooestrus versus non-prooestrus)). ANOVAs were followed by Scheffé’s post-hoc test, if appropriate. A p<0.05 was considered to be statistically significant.

## Results

### Influence of Presentation Mode, Genotype and Sex on the Investigation Duration

Investigation durations during sampling measured in the second experimental series with indirect sampling were significantly shorter than those measured in the first experimental series (direct sampling; **Tables 1** **and** **2**). The two-way ANOVA showed a significant effect of ‘genotype’ on the investigation duration during sampling for the two experimental series whereas a significant effect of ‘sex’ was revealed for the first experimental series only (first series: genotype: F(3,162) = 56.17, p<0.001; sex: F(1,162) = 40.95, p<0.001; interaction F(3,162) = 7.22, p<0.001; second series: genotype: F(3,158) = 33.64, p<0.001; sex: F(1,158) = 0.27, p = 0.60; interaction F(3,158) = 1.16, p = 0.33; **Tables 1** **and** **2**). Hyb2 mice investigated in both experimental series the juveniles significantly longer than all other genotypes (**Tables 1** **and** **2**). An interaction between genotype and sex was seen in the investigation duration in the first series only, where males investigated the juvenile significantly longer than females, except for C57BL/6JOlaHsd males (**Table 1** **)**.

**Table 1:** Raw investigation durations during sampling and choice for the first experimental series (sampling direct - choice direct), separately shown for all genotypes and sex of the experimental subjects (ES).

ES genotype	ES sex	Sampling	Choice (familiar juvenile)	Choice (unfamiliar juvenile)
129S1/SvImJ	Male	54.8±7.9 ^b^	25.4±2.8	40.1±4.4 ***
	Female	24.5±4.1 ^b’^	26.5±3.9	44.9±6.4 **
C57BL/6JOlaHsd	Male	41.6±3.8 ^b^	21.3±1.9	33.8±2.2 ***
	Female	45.8±3.0 ^b’^	17.6±1.9	25.3±2.4 **
Hyb1	Male	69.3±6.1 ^b^	28.6±3.5	44.9±4.3 **
	Female	39.5±5.1 ^b’^	24.2±2.7	37.3±4.2 **
Hyb2	Male	129.8±6.8 ^a^	56.5±2.7	74.7±5.0 **
	Female	81.5±7.5 ^a’^	34.7±4.8	65.4±6.1 ***

**Figure 2:**
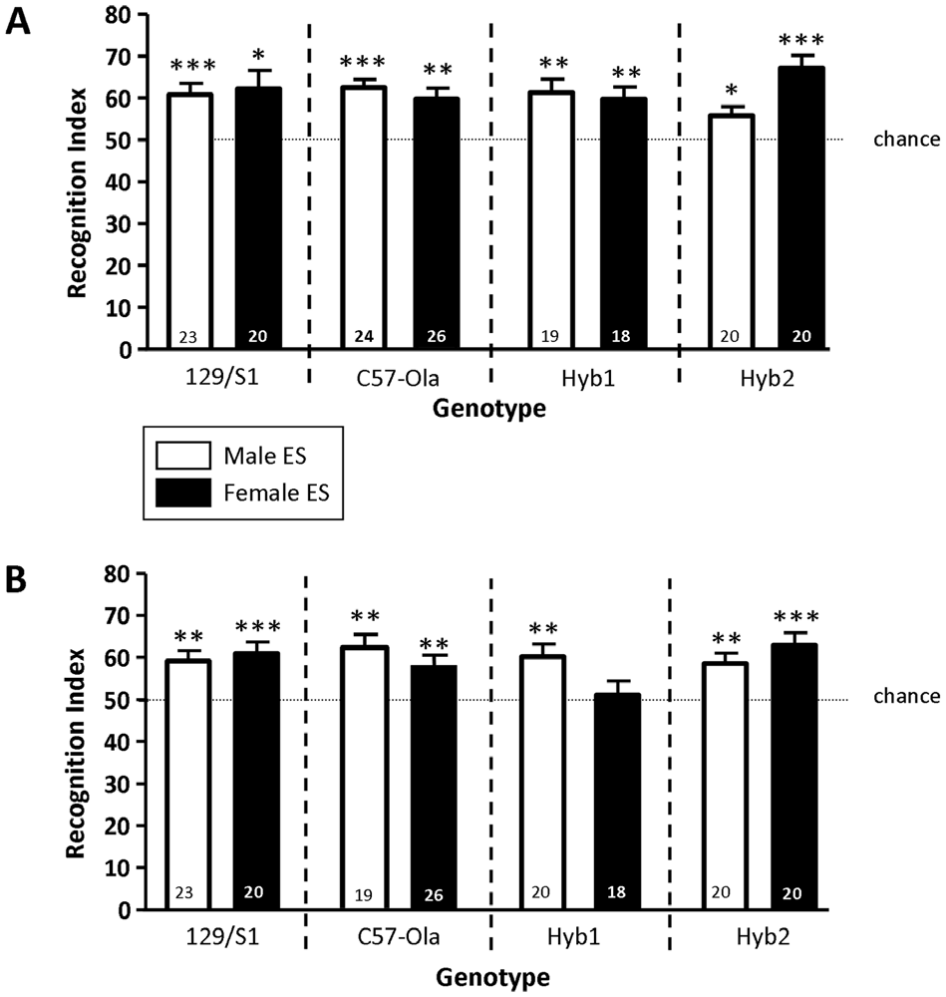
Recognition indices (RI; means+SEM) calculated from investigation durations during choice for the two experimental series. **A** shows that In the first experimental series the RIs of all tested groups [129S1/SvImJ (129/S1), Hyb1, Hyb2, C57BL/6JOlaHsd (C57-Ola)] of both male (white bars) and female ES (black bars) were significantly higher than 50 (chance) indicating an intact long-term recognition memory. **B** illustrates that in the second experimental series genotypes 129S1/SvImJ (both sex), Hyb2 (both sex), C57BL/6JOlaHsd (both sex), and Hyb1 males showed a significantly higher RI than 50 indicating an intact recognition memory. Hyb1 = F1-hybrid line (♀129S1/SvImJ×♂C57BL/6JOlaHsd), Hyb2 = F1-hybrid line (♀C57BL/6JOlaHsd×♂129S1/SvImJ), C57-Ola = C57BL/6JOlaHsd, 129/S1 = 129S1/SvImJ; numbers in bars = animals/group. * p<0.05, ** P<0.01 and *** P<0.001, one sample t-test *versus* 50.

Data are means ± SEM; for animals/group see Fig. 2A.

** p<0.01;

*** p<0.001 versus familiar juvenile in the same genotype and sex, t-test for repeated measures;

**a**: p<0.01 vs. **b**, **a’**: p<0.01 vs. **b’**, two-way ANOVA (genotype×sex) and Scheffé’s post-hoc test; significances shown for within sex comparism only.

**Table 2:** Raw investigation durations during sampling and choice for the second experimental series (sampling indirect (VFC) – choice direct), separately shown for all genotypes and sex of the experimental subjects (ES).

ES genotype	ES sex	Sampling	Choice (familiar juvenile)	Choice (unfamiliar juvenile)
129S1/SvImJ	Male	22.6±3.2 ^b^	24.1±2.3	33.7±2.8 **
	Female	18.9±3.1 ^d^	14.1±1.8	22.8±2.9 **
C57BL/6JOlaHsd	Male	21.3±2.7 ^b^	17.6±2.0	28.0±2.2 **
	Female	18.8±1.9 ^d^	20.1±1.52	29.0±3.1 **
Hyb1	Male	21.3±4.4 ^b^	20.5±1.9	31.4±3.5 **
	Female	30.2±5.2 ^e^	39.5±4.6	41.3±5.5
Hyb2	Male	55.0±3.5 ^a^	49.7±4.0	68.2±3.5 **
	Female	52.5±4.4 ^c^	20.0±3.4	31.0±5.1 ***

Data are means ± SEM; for animals/group see Fig. 2B.

* p<0.05;

** p<0.01;

*** p<0.001 versus familiar juvenile, t-test for repeated measures; **a**: p<0.01 vs. **b**, **c**: p<0.01 vs. **d**, **c**: p<0.05 vs. **e**, two-way ANOVA (genotype×sex) and Scheffé’s post-hoc test.

### Olfactory Recognition Performance

#### First series: Direct stimulus animal presentation during both sampling and choice

A two-way ANOVA did not show any differences in the RIs of any groups tested (genotype: F(3,162) = 0.09, p = 0.97; sex: F(1,162) = 0.82, p = 0.37; interaction: F(3,162) = 2.52, p = 0.06). As shown in **Figure 2A**, detailed analyses of the RIs using the one sample t-test revealed that mice from all genotypes and sex reached an RI significantly higher than chance level ( = 50) and, thus, showed an intact social recognition. This was confirmed by analysing the investigation durations during choice showing that all groups investigated the novel juvenile significantly longer than the previously encountered juvenile (paired t-test, **Table 1**).

#### Second series: Sampling performed in the VFC, direct stimulus animal presentation during choice

The two-way ANOVA did not show any statistically significant differences between the animals testing the RIs (genotype: F(3,158) = 1.44, p = 0.23; sex: F(1,158) = 0.72, p = 0.40; interaction: F(3,158) = 2.49, p = 0.06). As shown in **Figure 2B**, only Hyb1 females failed to recognize the previously exposed juvenile; all other groups showed a RI significantly higher than the chance level. This was confirmed by the statistical analyses of the investigation durations during choice. All – except female Hyb1– other groups tested spent significantly more time investigating the novel juvenile than the previously encountered juvenile (**Table 2**).

### Influence of Oestrus Cycle and Juvenile Sex on Recognition Ability

A two-way ANOVA testing the impact of prooestrus *versus* non-prooestrus on the memory performance in female mice revealed no significant differences in the RI in 129 (‘experimental series’: F(1,36) = 0.01, p = 0.97; ‘oestrus stage’: F(1,36) = 0.02, p = 0.87; interaction: F(1,36) = 0.21, p = 0.65) and Hyb1 females (‘experimental series’: F(1,28) = 0.10, p = 0.75; ‘oestrus stage’: F(1,28) = 0.78, p = 0.38; interaction: F(1,28) = 2.33, p = 0.14). Also for female C57BL/6JOlaHsd mice there was no significant impact of the oestrus cycle on the RI (‘experimental series’: F(1,48) = 0.93, p = 0.34; ‘oestrus stage’: F(1,48) = 0.39, p = 0.54; interaction: F(1,48) = 1.23, p = 0.27). Statistical analysis in Hyb2 females could not be performed because only one female was tested in prooestrus during both experimental series 1 and 2.

The results of the statistical analysis also indicated that the sex of the juvenile had no influence on the recognition performance in both experimental series (three-way ANOVA, first series: juveniles’ sex: F(1,95) = 0.81, p = 0,37; interaction between juveniles’ sex and genotype: F(2,95) = 1.65, p = 0.20; interaction between juveniles’ sex and experimental subjects’ sex: F(1,95) = 1.23, p = 0.27; second series: juveniles’ sex: F(1,109) = 0.16, p = 0,69; interaction between juveniles’ sex and genotype: F(2,109) = 0.32, p = 0.73; interaction between juveniles’ sex and experimental subjects’ sex: F(1,109) = 3.4, p = 0.07).

## Discussion

Here we investigated the social investigation and olfactory cued social recognition memory of 129S1/SvImJ and C57BL/6JOlaHsd mice and two hybrid lines obtained from the parental strains.

Compared to all other groups, the duration spent in direct social investigation was significantly increased in Hyb2, but not Hyb1 animals (**Table 1**), the former reared by female C57BL/6JOlaHSD, the latter by 129S1/SvImJ mice. At present, it remains purely speculative whether phenomena such as imprinting [34]–[36], maternal behaviour [37]–[39] and hybrid vigor ( = heterosis) [33] can explain the differences between our two hybrid strains. However, it is important to note that the situation seems very complex, since it appears to play a role which strain was used as the maternal and which other one as the paternal parental strain of the hybrids. Thus, further studies are needed to investigate the interaction between the possible contribution of these parameters on the expression of social investigation during adulthood towards conspecific juveniles in more detail.

All, but C57BL/6JOlaHsd, female mice spend less time investigating the juvenile during sampling than the males of the respective strain (**Table 1**). The fact that female animals show shorter investigation durations than males during direct encounters is well known from rats [40] and is – at least partially – here also confirmed for mice. Further studies have to investigate whether the genetic background plays indeed a role for the observed differences of the different sexes to engage in direct contact with the C57BL/6JOlaHsd juveniles. In any case, a reduced investigation duration during sampling (as seen in females of the three other strains) is not an indicative for a possibly impaired memory performance.

We measured in the VFC a significantly reduced investigation duration during sampling compared to the direct exposure. This implies that the stimulus animals were of less interest if only the volatile fraction of their olfactory signature was accessible in the VFC. It has been suggested that the non-volatile components of the olfactory signature’s trigger the intensive investigation of conspecifics in laboratory rodents [41]. Indeed, Keller et al. [42] found a decreased investigatory behaviour in animals with impaired vomeronasal organ resulting in an inability to acquire and process the non-volatile components of the ‘olfactory signature’. Therefore, the differences seen in the investigation durations in our study are likely to result from the inaccessibility of the non-volatile components of the conspecifics odour in the VFC apparatus. In addition, investigation durations during sampling were also affected by the genotype and sex of the experimental subjects. Hyb2 mice showed a significantly longer investigation of the juvenile compared to all other genotypes in both experimental series (see **Tables 1** and **2**). These data suggest that animals of mixed genetic background may show a higher social curiosity than those of their parental strains. This increased curiosity, however, has no predictive value of whether social recognition occurs or not. Indeed, animals that show a relatively low investigation duration during sampling successfully acquired for subsequent recognition (see **Table 1**, cf. [43]).

Authors of a recent study report an impaired short- and long-term social memory performance of male 129P2 mice, that was “rescued” by a (genetically engineered) reduced expression of the C-terminal Src kinase, reported to be causally involved in the proper development of the central nervous system and in the glutamatergic neurotransmission in the adult brain [19]. The authors conclude that social recognition is impaired in 129 animals [19]. In contrast, the data obtained in the first experimental series provide evidence for an unimpaired olfactory long-term recognition memory in both sexes and all genotypes tested in the social discrimination test **(** **Fig. 1A**). The main difference between our and the previously published data seems to be the experimental design of the behavioural test: The 129P2 mice were tested for their memory performance using the *social recognition procedure* in which during retrieval (‘choice’) the familiar and novel juvenile are not presented simultaneously, but in separate sessions. We tested using the *social discrimination task*, which provides a direct comparison of the investigation duration of the familiar and novel stimulus animal in the choice session. It seems that the social recognition procedure detected false negative results concerning the social recognition performance of animals of the 129 strain. Indeed, the reported ’abnormality’ of the 129 strain in various behavioural parameters [5]–[6], [44]–[45], which may have also contributed to the deficits reported for this strain in defined learning and memory tasks [1], [46], did not interfere in our paradigm of long-term social discrimination memory in either experimental series. To confirm that the 129S1/SvImJ animals used show a behavioural profile similar to that reported for 129 strains in defined behavioural paradigms, we submitted our animals to an open field test. Compared to C57BL/6JOlaHsd mice both males and females of the 129 strain showed the typical decreased motor activity, including a reduced time spent in running, rearing and a reduced number of lines crossed, and an increase in parameters indicating anxiety-like behaviour (see **Fig. S1** and **Table S1**). These observations match with the reported behavioural profile of the 129 strain in this test observed by some authors [2], [47]. Therefore, we hypothesize that the unimpaired long-term olfactory recognition memory monitored in our sample of 129S1/SvImJ mice represents the parent population of this strain concerning the performance in the social discrimination test.

The literature is inconsistent concerning the influence of the oestrus cycle on learning and memory performance: Some authors reported an improvement of memory performance during the prooestrus [30], [48]–[49], where others do not observe such an impact [50]–[51]. The differences were explained by oestrogen effects on different neuronal structures (and processes), [52]–[53] and a task specific impact of oestrogen [50]. Here, we failed to detect a statistically significant impact of the oestrus cycle on the social recognition performance in female mice of the two parental strains and Hyb1 during both experimental series. Although an analysis of the Hyb2 females could not be performed, the present data extend previous observations in rats [43] and suggest the robustness of the behavioural paradigm under study.

The data obtained in the second experimental series showed only for Hyb1 females an impaired social long-term recognition memory (see **Fig. 2B**). The observation that females and males of parental strains, 129S1/SvImJ and C57Bl/6OlaHsd, as well as the heterozygote Hyb2 strain and Hyb1 males showed an intact long-term recognition memory indicates that neither the genetical background of the parental strains nor the mixture of their genes is responsible for the recognition deficit of the Hyb1 females. Upon first view, the use of juveniles of the C57BL/6JOlaHsd strain as stimulus animals might provide a critical parameter that could have contributed to the observed effect. Indeed, it was reported that brain oxytocin signalling may underlie the intra- *versus* inter-strain social recognition in a procedure that involves the presentation of the stimulus animals in small cages [54]. However, there is no evidence for a difference in the oxytocinergic signalling between 129S1/SvImJ and C57Bl/6OlaHsd animals. Further, the fact that all other groups (including Hyb1 males and both genders of Hyb2) successfully recognized the previously presented juvenile in both experimental series renders a significant contribution of the strain origin of our stimulus animals rather unlikely. A more likely explanation for the observed differences might provide the challenge to match the volatile information from sampling with the complete olfactory signature collected during choice. Other than the protocol in which the experimental subjects get direct access to the stimulus animal’s body the outcome of the VFC testing is more challenging [24] and obviously more sensitive to the differences in the genetic background and/or sex. The inaccessibility to the non-volatile components, thought to provide the motivation for extensive investigatory behaviour [41], may increase the sensitivity to interfering effects that are difficult to exclude from this test procedure (e.g. handling procedures to place the juveniles in the VFC; see [26]). Therefore, testing in the VFC requires a particularly careful selection of control animals (e.g. wildtype) to avoid false positive or false negative interpretations.

Taken together, we show here that during direct encounter between the experimental subject and the stimulus animal(s) in the social discrimination procedure, mice of the 129S1/SvImJ strain and 129S1/SvImJ×C57BL/6JOlaHsd hybrids do not differ in their social recognition performance from C57BL/6JOlaHsd mice at an exposure interval of 24 h. If only the volatile components of the ‘olfactory signature’ are presented during sampling, female Hyb1 fail to show an intact long-term recognition memory. Thus, a social discrimination procedure based on direct exposure of the stimulus animals during *both* sampling and choice is suitable to investigate the relevance of distinct brain areas and/or intra- and extracellular signalling systems for long-term recognition memory. This procedure can be used with transgenic mouse mutants (including with a 129S1 genetic background), that may be corrupted in other learning and (long-term) memory tasks.

## Supporting Information

Figure S1 **Parameters obtained in the open field of the different genotypes and sex (white bars: male; black bars: female; means+SEM).** **A** illustrates the time spent in the inner, unprotected part of a 1 m×1 m wide open field with 50 cm high walls. **B** shows the numbers of lines crossed (of a virtual grid of 10 cm×10 cm) in the open field. Two-way ANOVA (genotype×sex) followed by Scheffé‘s post-hoc test. a: p<0.01 vs. b, b’ and b’’, a’: p<0.05 vs. b’’, Hyb1 = F1-hybrid line (♀129S1/SvImJ×♂C57BL/6JOlaHsd), Hyb2 = F1-hybrid line (♀C57BL/6JOlaHsd×♂129S1/SvImJ), C57-Ola = C57BL/6JOlaHsd, 129/S1 = 129S1/SvImJ; numbers in bars = animals/group.
(PPT)

Table S1 **Time the experimental subjects spent grooming and rearing in the open field (means ± SEM; n = 18–20 per group).**
(DOC)
