# Supplementary material for: Social Investigation and Long-Term Recognition Memory Performance in 129S1/SvImJ and C57BL/6JOlaHsd Mice and Their Hybrids
Source: PLoS One. 2013 Jan 16;8(1):e54427. doi: 10.1371/journal.pone.0054427 (PMC3546984; doi:10.1371/journal.pone.0054427)
Supplement: Figure S1 — Parameters obtained in the open field of the different genotypes and sex (white bars: male; black bars: female; means+SEM). A illustrates the time spent in the inner, unprotected part of a 1 m×1 m wide open field with 50 cm high walls. B shows the numbers of lines crossed (of a virtual grid of 10 cm×10 cm) in the open field. Two-way ANOVA (genotype×sex) followed by Scheffé‘s post-hoc test. a: p<0.01 vs. b, b’ and b’’, a’: p<0.05 vs. b’’, Hyb1 = F1-hybrid line (♀129S1/SvImJ×♂C57BL/6JOlaHsd), Hyb2 = F1-hybrid line (♀C57BL/6JOlaHsd×♂129S1/SvImJ), C57-Ola = C57BL/6JOlaHsd, 129/S1 = 129S1/SvImJ; numbers in bars = animals/group. (PPT) [file pone.0054427.s001.ppt]

## Slide 1
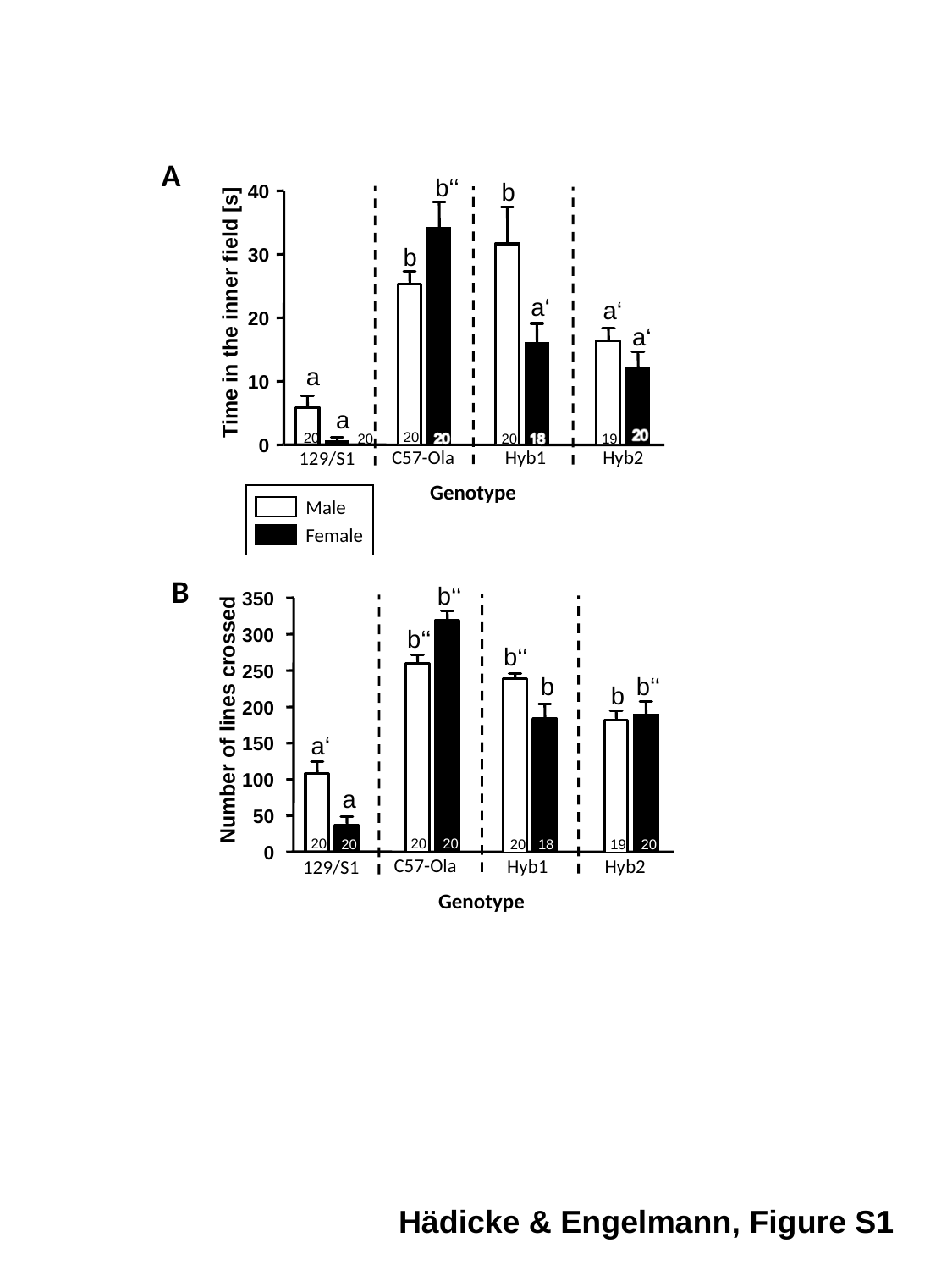

A
b‘‘
b
20
C57-Ola
b
40
30
a‘
a‘
Time in the inner field [s]
20
a‘
a
10
a
20
20
20
19
0
Hyb1
Hyb2
129/S1
Genotype
Male
Female
B
b‘‘
b‘‘
20
20
C57-Ola
350
b‘‘
b
b‘‘
b
20
18
19
20
Hyb1
Hyb2
300
250
200
Number of lines crossed
a‘
150
100
a
50
20
20
0
129/S1
Genotype
Hädicke & Engelmann, Figure S1
