# Supplementary material for: Social Investigation and Long-Term Recognition Memory Performance in 129S1/SvImJ and C57BL/6JOlaHsd Mice and Their Hybrids
Source: PLoS One. 2013 Jan 16;8(1):e54427. doi: 10.1371/journal.pone.0054427 (PMC3546984; doi:10.1371/journal.pone.0054427)
Supplement: Table S1 — Time the experimental subjects spent grooming and rearing in the open field (means ± SEM; n = 18–20 per group). (DOC) [file pone.0054427.s002.doc]

**Table S1.** Time the experimental subjects spent grooming and rearing in the open field (means ± SEM; n = 18-20 per group).

| Genotype | Sex | Grooming [s] | Rearing [s] |
| --- | --- | --- | --- |
| 129S1/SvImJ | Male | 4.84 ± 1.60 ^a^ | 12.95 ± 3.07 ^a^ |
|  | Female | 6.34 ± 1.52 ^a’^ | 3.74 ± 1.43 ^a^ |
| C57BL/6JOlaHsd | Male | 7.21 ± 0.90 ^a’^ | 72.62 ± 4.17 ^c^ |
|  | Female | 5.95 ± 0.70 ^a^ | 59.51 ± 2.67 ^b^ |
| Hyb1 | Male | 11.16 ± 1.80 ^a’^ | 55.72 ± 2.95 ^b’^ |
|  | Female | 11.85 ± 1.45 ^a’^ | 44.23 ± 6.37 ^b^ |
| Hyb2 | Male | 16.80 ± 4.28 ^a’’^ | 48.33 ± 5.09 ^b’^ |
|  | Female | 8.22 ± 1.65 ^a’^ | 46.09 ± 5.20 ^b’^ |

a: p < 0.05 vs. a’’, b, b’ and c; b: p < 0.01 vs. c; two-way ANOVA (genotype x sex) and Scheffé‘s post-hoc test. Hyb1 = F1-hybrid line (♀129S1/SvImJ x ♂C57BL/6JOlaHsd), Hyb2 = F1-hybrid line (♀C57BL/6JOlaHsd x ♂129S1/SvImJ).
